# Statin use and low-density lipoprotein cholesterol target achievement for primary prevention of atherosclerotic cardiovascular disease in patients with type 2 diabetes mellitus: a multicenter cross-sectional study in Sri Lanka

**DOI:** 10.1371/journal.pone.0319030

**Published:** 2025-02-21

**Authors:** P.D.W.D. De Zoysa, T.P. Weerarathna, I.L.A.N. Darshana, K.G.P. Wasana, B. Piyasekara, M.M.P.T. Jayasekara, V. Sujanitha, S. Silva, C. Mettananda, G.U. Ramadasa, L.P.M.M.K. Pathirage

**Affiliations:** 1 Faculty of Medicine, University of Ruhuna, Sri Lanka; 2 Faculty of Health Sciences, CINEC Campus, Sri Lanka; 3 Diabetes Center, Co-operative Hospital, Galle, Sri Lanka; 4 Faculty of Medicine, General Sir John Kotelawala Defense University, Sri Lanka,; 5 Faculty of Medicine, University of Jaffna, Sri Lanka,; 6 Faculty of Medicine, University of Sri Jayewardenepura, Sri Lanka,; 7 Faculty of Medicine, University of Kelaniya, Sri Lanka,; 8 Faculty of Medicine, University of Sabaragamuwa, Sri Lanka,; 9 Faculty of Medicine, University of Peradeniya, Sri Lanka; Kerman University of Medical Sciences Physiology Research Center, IRAN, ISLAMIC REPUBLIC OF

## Abstract

**Background:**

Statin therapy serves a crucial role as a primary preventive strategy against atherosclerotic cardiovascular disease (ASCVD) in patients with type 2 diabetes mellitus (T2DM). Even though diabetes poses a significant and growing health concern in Sri Lanka, there is a lack of information regarding the prevalence and intensity of statin prescriptions and the achievement of recommended LDL-C targets in diabetic patients for the primary prevention of ASCVD within the nation. We aimed to assess the prevalence and intensity of statin prescriptions, target LDL-C achievement, and factors associated with target LDL-C achievement for the primary prevention of ASCVD in T2DM patients across several tertiary care facilities in Sri Lanka.

**Methods:**

A multi-centered, cross-sectional study was conducted among T2DM patients without clinical ASCVD attending six tertiary care medical clinics in Sri Lanka. Data on ASCVD risk factors and statin prescription were collected using an interviewer-administered questionnaire. ASCVD risk was calculated using the WHO charts. Atorvastatin 20 mg/ rosuvastatin 10 mg was defined as high-intensity statins and target LDL-C was defined as < 70 mg/dL for moderate to high and < 100 mg/dL for low-risk groups of ASCVD according to the NICE guideline. The independent sample t test, one-way ANOVA and chi-square test were used for data analysis as appropriate. Factors linked to achieving LDL-C targets were determined through multiple logistic regression analysis. Level of significance was considered as 0.05.

**Results:**

Of the 2013 participants studied, 46.7% were at moderate-high risk and the rest were at low risk of ASCVD. All were eligible for statin therapy, and 84.1% were prescribed statins. High-intensity statins had been prescribed only for 38.5% of moderate-high-risk patients. Nonetheless, high-intensity statins have also been prescribed for 30.7% of low-risk patients. LDL-C target achievement was studied in a randomly selected subsample of 683 and 65.4% (70.7% in low-risk patients and 60.3% in moderate-high-risk patients) achieved LDL-C targets. Of moderate-high-risk patients, 46.3% had not achieved target LDL-C even with high-intensity statin therapy. Female gender (OR = 1.52, 95% CI 1.03-2.24, p = 0.036), poor adherence to statins (OR = 1.67, 95% CI 1.18-2.37, p = 0.004), poor glycemic control (OR = 2.27, 95% CI 1.41-3.65, p = 0.001), and inadequate physical activity (OR = 1.48, 95% CI 1.04-2.10, p = 0.031) were significantly associated with failing to achieve LDL-C targets.

**Conclusion:**

Only about one third of diabetes patients with moderate-high ASCVD risk received high-intensity statins. Even with high-intensity statin therapy, nearly half of the treated patients failed to meet recommended LDL-C targets.

## Introduction

Type 2 diabetes mellitus (T2DM) is a major global public health concern, owing to its rising incidence and related complications. Currently, more than 10.5% of the adult population worldwide lives with this condition, and projections indicate that this number will rise by 25% in 2030 and 51% in 2045 [1,2]. The incidence of T2DM across the globe is increasing due to a variety of factors, including lifestyle changes, urbanization, and demographic transition [3,4]. The disease is associated with numerous complications, including cardiovascular diseases and microvascular complications, thereby increasing morbidity and mortality [5]. There is a significant economic impact, affecting healthcare systems around the world due to the financial expenditures associated with treatment of the disease and management of its complications [6,7]. The situation is equally alarming in Sri Lanka. Studies have shown that the prevalence of T2DM is highest in Sri Lanka among Southeast Asia, where one in four Sri Lankan adults has the disease, and its prevalence has tripled over the last three decades [8,9]. The rising prevalence of diabetes in Sri Lanka is attributed to several factors including genetic predisposition, cultural, demographic, and environmental changes [10,11].

Atherosclerotic cardiovascular disease (ASCVD) includes coronary artery disease (CAD), cerebrovascular disease (CVD), or peripheral artery disease (PAD) and constitutes a major complication of T2DM. Various additional risk factors, including dyslipidemia, hypertension, tobacco use, a familial predisposition to early cardiovascular disease, and advancing age, contribute to an elevated risk of ASCVD [12]. Nevertheless, numerous studies, inclusive of meta-analysis, have shown that diabetes is an independent risk determinant for ASCVD after accounting for all other factors [12–14]. Furthermore, the prevalence of CAD in patients with T2DM is two to three times higher than in the non-diabetic population [12]. In 2021, CAD emerged as the primary cause of mortality worldwide and ranked as the second leading contributor to disability-adjusted life years (DALYs) for both sexes [15]. In Sri Lanka, the mortality rates for CAD and CVD reached alarming levels of 5.58 and 6.78 per 100 cases in 2019, while they accounted for one-third of the total health expenditure in 2017 and 2018 according to the annual health bulletin 2021 and Ministry of Health national health accounts 2017 and 2018 [16]. Consequently, Sri Lanka is confronted with the dual epidemic of T2DM along with its primary complication, ASCVD, as well as the associated economic repercussions.

Therefore, the primary prevention of ASCVD is far more cost-effective than managing an already established condition, both in practical and financial terms. Individuals with T2DM are more likely to have lipid abnormalities, increasing their risk of developing ASCVD. The evidence supporting the reduction of low-density lipoprotein cholesterol (LDL-C) levels in the primary prevention of ASCVD among individuals with T2DM is thoroughly established [17,18]. Statins are the drug of choice in reducing LDL-C levels in the primary and secondary prevention of ASCVD among patients with T2DM [19–22]. Additional cardiovascular benefits, such as improved endothelial function, anti-inflammatory effects, and plaque stabilization, complement the lipid-lowering effects of statins and collectively reduce the risk of ASCVD [23,24]. Guidelines recommend two statin dosing intensities (high intensity and moderate intensity) for individuals with diabetes depending on their ASCVD risk for primary prevention [25,26].

Despite the guidelines, endorsing statin use based on ASCVD risk to reduce cardiovascular morbidity and mortality among individuals with T2DM, adhering to these recommendations can be challenging. A research analysis involving 28,807 patients across a contemporary healthcare system revealed that only two-thirds of intermediate and high-risk diabetes patients were undergoing guideline-recommended statin intensity therapy at the five-year follow-up, highlighting a considerable lack of adherence to established recommendations [27]. Furthermore, in a real-world primary ASCVD prevention cohort with 282,298 diabetic patients, more than one-third of statin-eligible patients were not prescribed statin therapy [28]. Only 62.7% of eligible diabetic patients in low middle income countries (LMICs) like Ethiopia received statin treatment, and only 15.6% of those in the high-risk group received recommendations for high-intensity therapy, according to a recent study [29]. A recent Indian study indicated that while over 95% of people with T2DM were receiving statin therapy, only 25% managed to achieve the target LDL cholesterol level of under 100 mg/dL [30]. Research in Sri Lanka shows a considerable gap concerning the role of statins in the primary prevention of ASCVD among those with T2DM. A study focusing on both primary and secondary prevention of ASCVD in T2DM reveals more than 90% were on statins. Nonetheless, it indicates that the prescribing of statins at the correct intensity based on risk categories falls significantly short of the recommended guidelines [31]. Sparsity of appropriate health care policies for primary prevention of ASCVD in Sri Lanka may also be a reason for this deficiency. Addressing these gaps is crucial to improving the cardiovascular outcomes in the T2DM population in Sri Lanka.

The objectives of this multicenter study were to assess the rates of statin prescription practices and target LDL-C achievements for the primary prevention of ASCVD in patients with T2DM across various tertiary care settings in Sri Lanka. This study aimed to fill existing knowledge gaps regarding the use of statins in the primary prevention of ASCVD in T2DM patients within the Sri Lankan healthcare context.

## Methods

### Study design and study setting

A hospital based multicenter study was conducted among T2DM patients during January and April 2024 following the STROBE statement. A cross-sectional study with an analytical component was selected as the study design to obtain a snapshot of ASCVD risk, statin use, and LDL-C target achievement with identification of its associated factors among those patients. The study selected six tertiary care hospitals from five main provinces in Sri Lanka: National Hospital Galle in the Southern province; Colombo South Teaching Hospital; and University Hospital of Kotelawala Defense University in the Western province; Teaching Hospital Peradeniya in the Central province; and Teaching Hospital Jaffna in the Northern province, and Rathnapura Teaching Hospital in the Sabaragamuwa province. The ethical clearance for the study was granted by the Ethics Review Committee, Teaching Hospital Karapitiya, Sri Lanka (THK/ERC/23/12). Before the commencement of the study, all the study subjects signed a written informed consent.

### Study population and study sample

The individuals diagnosed with T2DM in Sri Lanka were selected as participants for this study. The study included a sample of individuals aged between 40 and 70 years who had been diagnosed with T2DM by a physician and had been regularly followed up at outpatient medical clinics for a minimum of six months in any of the selected six tertiary care hospitals of the country.

The pregnant and lactating females, patients with clinically established ASCVD, patients on lipid lowering medications other than statins, patients with a total cholesterol level of 8 mmol/L or more, and an eGFR of <  60 mL/min/1.73 m^2^ were excluded from the study. Statins are not recommended during pregnancy, and most patients who are breastfeeding are also advised against using statins for the primary prevention of ASCVD. Individuals with extremely high total cholesterol levels of 8 mmol/L or greater, and an eGFR of less than 60 mL/min/1.73 m², were excluded from the study for two main reasons. Firstly, they should be prescribed statins regardless of their cardiovascular risk status, and secondly, they met the exclusion criteria based on the risk prediction chart utilized (WHO risk chart).

The study’s sample size was determined using the formula provided by Lwanga and Lemeshow (1991), which is designed for sample size calculations based on proportions, at a significance level of 0.05 and a precision degree of 2.5%. This calculation took into account an expected population proportion of 11.6% for LDL-C target achievement and a 10% non-response rate [32]. The minimum required sample size was 668 patients. However, using multistage sampling, the study’s early phase recruited a total of 2013 patients from six tertiary care hospitals across five provinces. There are about 1200 patients registered in a routine clinic and we wanted to enroll nearly 400 from each clinic. From these chosen six study centers, patients were selected using a systematic random sampling technique with a skip interval of four (1200/ 400). The individuals diagnosed with T2DM who were registered at the medical clinic across six centers were included on the days designated for data collection, with every third participant according to the register (irrespective of gender) selected as a study subject following the established inclusion and exclusion criteria. In the next step, 683 individuals were selected using the simple random sampling method among the patients who were on statins to assess LDL-C target achievement, and this exceeded the calculated minimum sample size.

### Data collection

Data was gathered through a semi-structured questionnaire administered by an interviewer, encompassing both sociodemographic and disease-related information. Relevant clinical information was extracted from the clinic records available with the patient. The 10-year risk of a fatal or non-fatal cardiovascular event by gender, age, systolic blood pressure, total blood cholesterol, and smoking status in patients with T2DM was assessed using the updated WHO risk chart for Southeast Asia [33]. Trained pre-intern medical officers collected all the data from all study centers. They were trained in data collection, including data extraction from medical records, blood pressure monitoring, and obtaining anthropometric measurements by the principal investigator.

Among the patients on statins, a subsample was selected to assess LDL-C target achievement. In this subsample, the status of blood pressure control and glycemic control as well as physical activity level and drug adherence were assessed. After five minutes of the patients being seated, the systolic and diastolic blood pressures (SBP/DBP) were assessed using a compact and fully automatic blood pressure monitor (OMRON HEM-7120), with the average of three consecutive readings recorded. The subsample’s LDL-C level and glycemic control were evaluated. In the subsample, glycosylated hemoglobin (HbA_1C_) levels were measured to evaluate glycemic control, while LDL-C levels were analyzed to determine if LDL-C targets were met. Biochemical investigations were conducted on the recruited individuals, including the measurement of lipid profile markers [triglycerides (TG), total cholesterol (TC), and high-density lipoprotein cholesterol (HDL-C) levels] and HbA_1C_. High-performance liquid chromatography with a fully automated analyzer (BIORAD D analyzer, USA) was used to assess HbA_1C_. Using spectrophotometric principles, lipid profile parameters were determined using a fully automated analyzer (Huma star − 600 HS- Germany). LDL-C was calculated using the Friede-Wald equation. Medication adherence for statin therapy was assessed using the validated Sinhala version of the Morisky Medication Adherence Scale [34]. Physical activity level was assessed based on the validated Sinhala version of the International Physical Activity Questionnaire (IPAQ) [35].

### Operationalization of variables

The use of atorvastatin 20 mg or rosuvastatin 10 mg was considered a high-intensity statin according to the NICE guidelines 2023 [26]. ASCVD risk was assessed based on the WHO risk chart for Southeast Asia [33]. The ASCVD risk was categorized as low (<10%), moderate (10%-20%), and high (>20%) based on the risk assessment derived from the WHO chart. According to ADA 2024, hypertension is defined by a SBP > 130 mmHg or a DBP > 80 mmHg, and for those receiving treatment, the target blood pressure should be below 130/80 mmHg if it can be safely attained [25]. Recommended LDL-C target for higher ASCVD risk was considered as less than 70 mg/dL (<70 mg/dL) for moderate and high ASCVD risk groups while less than 100 mg/dL (<100 mg/dL) for low ASCVD risk group according to ADA 2024 [25]. If the HbA_1C_ level was less than seven percent (<7%), it was considered as having optimal glycemic control according to ADA 2024 and NICE 2023 [36]. Having a minimum of 3000 MET minutes per week passed on IPAQ was considered as physically active [35]. Social class was categorized according to the social class categorization given by Baker and Hall in Practical Epidemiology [37]. Social class I and II were considered the higher social class.

### Data analysis

Data was analyzed using SPSS software (25^th^ Version). A normality assessment of the data set was done before the analysis using the Anderson-Darling test. Missing values were treated by data imputation with mean. Descriptive statistics were applied for the data set in terms of mean ± standard deviation (SD) and frequency (percentages) as appropriate. The independent sample t-test was used to compare two means (Mean LDL-C levels according to type (atorvastatin vs rosuvastatin) and intensity (low vs high) of statins. More than two means (Mean LDL-C level according to ASCVD risk (low vs moderate vs high) were compared using one-way ANOVA following mean separation with Tukey’s test. The Chi-square test was used to compare categorical variables. The Chi-square test was applied to identify factors associated with achieving the LDL-C target. Achieving the LDL-C target (target achieved or not) was considered as the dependent variable. Socio-demographic factors (gender, age, educational level, occupational status, income, social class, living status, and marital status), disease-related factors (age of the diagnosis of T2DM, duration of T2DM, and glycemic control status), treatment-related factors (type of statin, intensity of statin dose and drug adherence), other possible associated factors such as family history of T2DM or premature ASCVD, ASCVD risk, BP control status, and physical activity level were considered as independent variables. Factors with p value less than 0.2 were identified and used as independent variables for the model in multiple logistic regression in order to control potential confounders. The results were presented using an odds ratio (OR) with a 95% confidence interval (CI). Level of significance was considered 0.05 for all the analysis.

## Results

The average age of the entire study sample (n =  2013) was 58.0 ±  8.6 years, with the female preponderance 60.5% (n =  1218). Study subjects have been diagnosed with T2DM at the age of 47.8 ±  10.7 years. The total duration of T2DM up to the study date was 10.2 ±  8.4 years. The family history of T2DM in a first-degree relative was noted in 58.3% (n = 1173) while a family history of premature coronary artery disease in a first-degree relative was detected in 24.1% (n = 241). Other baseline characteristics of the sample including sociodemographic information are presented in Table 1.

**Table 1:** Baseline characteristics of the study sample (n = 2013).

Variable	Results
Number (%) of female gender	1218 (60.5)
Number (%) of T2DM patients according to ethnicity	
Sinhala	1561 (77.5)
Others (Tamil, Muslim, Burger)	452 (22.5)
Number (%) of T2DM patients according to age group	
Between 40 and 60 years	1157 (57.5)
Above 60 and up to 70 years	856 (42.5)
Mean ± SD age (years)	58.0 ± 8.6
Number (%) of patients according to age at the diagnosis of T2DM	
Before 40 years	484 (24.0)
40 and above years	1529 (76.0)
Mean ± SD age at the diagnosis of T2DM (years)	47.8 ± 10.7
Number (%) of patients according to duration of the T2DM	
Less than 10 (years)	1265 (62.8)
10 and above (years)	748 (37.2)
Mean ± SD duration of the T2DM	10.2 ± 8.4
Number (%) of patients according to employment status	
Employed in a regular job	781 (38.7)
Unemployed	1232 (61.3)
Number (%) of patients according to social class	
Social Class I & II	177 (8.8)
Social Class III, IV and V	1836 (91.2)
Number (%) of patients according to educational level	
Not completed secondary education	822 (48.9)
Completed secondary education	1191 (59.1)
Number (%) of patients according to monthly income (LKR)	
Satisfactory (50,000 and above)	339 (16.8)
Unsatisfactory (below 50,000)	1674 (83.2)
Number (%) of patients with comorbidities (Other than dyslipidemia)	
Yes	1346 (66.8)
Hypertension	789 (58.6)
Liver disease	45 (3.3)
Asthma	80 (5.9)
Multiple responses	432 (32.1)
BP Control status	
Optimal Control achieved (BP < 130/80 mmHg)	1442 (71.6)
Optimal control not achieved	571 (28.4)
Mean ± SD SBP (mmHg)	132.3 ± 20.3
Mean ± SD DBP (mmHg)	78.9 ± 10.8

BP; blood pressure, DBP; diastolic blood pressure, SBP; systolic blood pressure, T2DM; type 2 diabetes mellitus.

Nearly half of the sample had low ASCVD risk (53.3%) while moderate and high risk were detected in 38.8% and 7.9% respectively. Statins were prescribed for 84.4% of the total sample (80.9% in low-risk individuals, 88.1% in those with moderate risk, and 85.6% in high-risk patients, respectively). Statin prescriptions were significantly higher (p < 0.001) in patients with moderate-high ASCVD risk (87.7%) as compared to those with the low-risk category (80.9%). Most (96.7%) of the patients were treated with atorvastatin while the rest were treated with rosuvastatin. High-intensity statin had been prescribed only for 37.4% of patients in the moderate-high-risk group. Statin usage and type of statin significantly differed according to ASCVD risk category. However, there was no significant association between ASCVD risk and intensity of statin dose (Table 2).

**Table 2:** ASCVD risk and statin usage.

	ASCVD risk (n = 2013)			Total	Test statistic^#^ (df = 2) p-value
	Low 1072(53.3%)	Moderate 781(38.8%)	High 160(7.9%)		
**Statin use** (**n = 2013)**					
***Yes***	868(51.3%)	688(40.6%)	137(8.1%)	1693(100.0%)	17.44 <0.001*
***No***	204(63.7%)	93(29.1%)	23(7.2%)	320(100.0%)	
**Type of statin (n = 1693)**					
Atorvastatin	851(51.9%)	660(40.2%)	129(7.9%)	1640(100.0%)	9.25 0.010*
Rosuvastatin	17(32.1%)	28(52.8%)	8(15.1%)	53(100.0%)	
**Intensity of statin (n = 1693)**					
Low	539(50.9%)	442(41.7%)	78(7.4%)	1059(100.0%)	3.85 0.251
High	329(51.9%)	236(38.8%)	59(9.3%)	634(100.0%)	

ASCVD; Atherosclerotic cardiovascular disease. * Statistically significant at 0.05 significance level.

### #chi square test.

**Fig 1:**
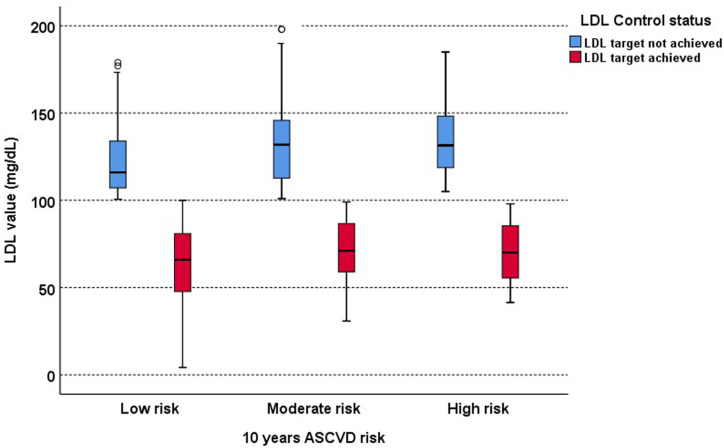
Comparison of LDL-C levels according to 10-year ASCVD risk (n  =  683) and LDL target achievement.

LDL-C target achievement was assessed in a sub-sample of 683 patients with T2DM. The mean LDL-C level was significantly different according to the ASCVD risk category. Post hoc analysis revealed that the mean LDL-C level was significantly lower in the low-risk group compared to moderate (p < 0.001) or high (p < 0.001) risk groups but no significant difference between moderate and high-risk groups (p = 0.640). The patients who achieved the LDL-C target had statistically significantly lower mean LDL-C levels in all three ASCVD risk groups (p < 0.001) (Fig 1). There was no statistically significant difference in mean LDL-C level in any of the ASCVD risk groups according to type of statin (p =  0.904) or intensity of statin (p =  0.572).

**Fig 2:**
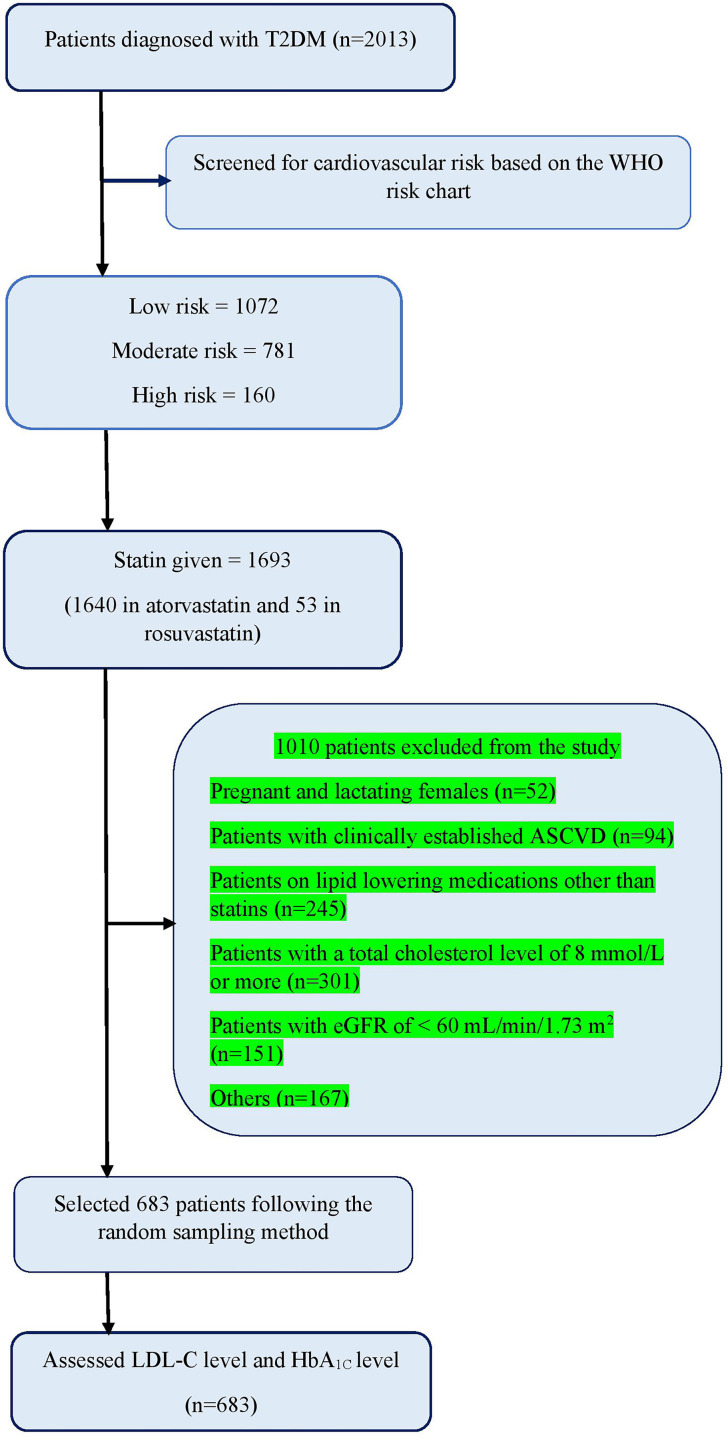
Study flow diagram. HbA_1C_; glycated hemoglobin, LDL-C; low-density lipoprotein cholesterol, T2DM; type 2 diabetes mellitus, WHO; World Health Organization.

High-intensity statin had been recommended only for 38.5% (134/348) of patients in the moderate- high-risk group, but among them, 46.3% (n = 62) have not achieved the target LDL-C. However, high-intensity statin also had been recommended for 31.9% (107/335); of patients in the low-risk group, among them 73.8% (n = 79) had achieved the target LDL-C. The LDL-C target achievement was noted by 70.7% (n = 237) in the low-risk group, 62.6% (n = 181) in the moderate-risk group, and 49.2% (n = 29) in the high-risk group respectively (Table 3). The LDL-C target achievement for the total group was 65.4% (n = 447). The detailed study summary is shown in Fig 2.

**Table 3:** Statin usage and LDL-C target achievement (n = 683).

	LDL-C target achievement		Total n = 683
	Achieved (n = 447)	Not achieved (n = 236)	
**ASCVD Risk**			
Low	237(70.7%)	98(29.3%)	335(100.0%)
Moderate	181(62.6%)	108(37.4%)	289(100.0%)
High	29(49.2%)	30(50.8%)	59(100.0%)
**Type of statin**			
Atorvastatin	432(65.5%)	228(34.5%)	660(100.0%)
Rosuvastatin	15(65.2%)	8(34.8%)	23(100.0%)
**Intensity of statin**			
Low	296(67.0%)	146(33.0%)	442(100.0%)
High	151(62.7%)	90(37.3%)	241(100.0%)

### LDL-C; low-density lipoprotein cholesterol.

In the univariate analysis gender(p = 0.020), glycemic control status (<0.001), medication adherence to statins (p = 0.001), ASCVD risk level (p = 0.004) and physical activity level (p = 0.010) had statistically significant association with LDL-C target achievement while age(p = 0.678), educational level(p = 0.836), occupational status(p = 0.818), income(p = 0.459), social class(p = 0.186), living status(p = 0.880), duration of T2DM(p = 0.693), type of statin(p = 0.981), intensity of statin(p = 0.257), family history of T2DM(p = 0.298) or premature ASCVD(p = 0.270), and BP control status of the patients(p = 0.952) were failed to identify a significant association with LDL-C target achievement. However, all the factors which had significance value less than 0.20 in univariate analysis were included in the multiple logistic regression. Multiple logistic regression revealed that females had 1.52 times likely risk compared to males (OR = 1.52, 95% CI 1.03-2.24), poor adherence to statin therapy had 1.67 times likely risk compared to patients with good adherence to statin therapy (OR = 1.67, 95% CI 1.18-2.37), uncontrolled glycemic status had 2.27 times likely risk compared to patients with controlled glycemic status (OR = 2.27, 95% CI 1.41-3.65), and being physically less active according to IPAQ scale had 1.48 times likely risk compared to physically active patients (OR = 1.48, 95% CI 1.04-2.10) for not achieving LDL-C target. Interestingly, it was found that patients with low ASCVD risk were 0.58 times more likely to achieve the LDL-C target (OR = 0.58, 95% CI 0.42–0.81) compared to moderate and high ASCVD group while social class failed to emerge as either a significant risk or protective factor (Table 4).

**Table 4:** Factors associated with patients achieving the LDL-C target (n = 683).

				Univariate analysis	Multivariate analysis	
	**LDL-C Target**		**Total** **n (%)**	**Adjusted OR** **(95%CI)**	**Adjusted OR** **(95%CI)**	**p-value**
	**Achieved** **n(%)**	**Not achieved** **n(5%)**				
**Gender**						
Female	307(62.8%)	182(37.2%)	489(100.0%)	1.54 (1.07 -2.21)	1.52 (1.03 -2.24)	0.036 *
Male (R)	140(72.2%)	54(27.8%)	194(100.0%)			
**Social Class**						
Lower	410(64.8%)	223(35.2%)	633(100.0%)	1.55 (0.81 -2.97)	1.13 (0.56 -2.24)	0.737
Higher (R)	37(74.0%)	13(26.0%)	50(100.0%)			
**Adherence to statin treatment**						
Unsatisfactory	121(56.5%)	93(43.5%)	214(100.0%)	1.75 (1.25 -2.45)	1.67 (1.18 -2.37)	0.004 *
Satisfactory (R)	326(69.5%)	143(30.5%)	469(100.0%)			
**Glycemic control status**						
Uncontrolled	337(61.6%)	210(38.4%)	547(100.0%)	2.64 (1.66 -4.18)	2.27 (1.41 -3.65)	0.001 *
Controlled (R)	110(80.9%)	26(19.1%)	136(100.0%)			
**Physical activity level**						
Inactive	262(61.8%)	162(38.2%)	424(100.0%)	1.55 (1.11 -2.16)	1.48 (1.04 -2.10)	0.031 *
Active (R)	185(71.4%)	74(28.6%)	259(100.0%)			
**ASCVD risk**						
Low	237(70.7%)	98(29.3%)	335(100.0%)	0.63 (0.46 -0.87)	0.58 (0.42 -0.81)	0.001 *
Moderate/high (R)	210(60.3%)	138(39.7%)	348(100.0%)			

LDL-C; low-density lipoprotein cholesterol, (R); reference. * Statistically significant at 0.05 significance level.

## Discussion

This multicenter study involving 2013 patients with T2DM, carried out across six tertiary care facilities in five provinces of Sri Lanka, investigated the use of statins and the achievement of recommended therapeutic targets for LDL-C, and revealed several significant insights.

Overall, 84% of participants in this study have been prescribed statins as a primary prevention measure, and it is a commendable finding in a country with an escalating burden of T2DM and ASCVD. While the percentages of patients prescribed statins were quite similar across the three ASCVD risk categories (80.9% for low risk, 88.1% for moderate risk, and 85.6% for high risk), there was a significantly higher statin usage (p < 0.001) in the moderate-high risk group (87.7%) when compared to the low-risk group (80.9%), which is also encouraging observation. The findings from this study showed a more positive trend than the data from a recent African meta-analysis, where only 48.82% of eligible individuals with T2DM had been prescribed statin [38]. Another study from India revealed that only half of the clinic-based patients in India with T2DM had received statins, and the high-intensity statin prescription was very low and almost the same in all risk groups: high-risk (14.5%), medium-risk (11.8%), and low-risk (13.5%) (p =  0.31) [39]. A study from the United States involving more than 241,232 patients from 90 centers reported stain use only in 65% of patients with diabetes, with high-intensity statins being prescribed in 58% [24].

Although nearly half of the study participants in our study (46.3%) were in either moderate or high ASCVD risk categories, high-intensity statins were prescribed only for 38.5% of the patients. As a result of inappropriate dosing, mean LDL-C levels were significantly higher in the groups with both moderate and high ASCVD risk who have not achieved the therapeutic LDL-C target compared to the low ASCVD risk category. In contrast, 329 patients out of 1072 in the low ASCVD risk category were unnecessarily prescribed high-intensity statins. Inappropriate statin dosing despite higher overall statin use among patients with T2DM has been reported in another study from Sri Lanka. They reported that even though 93% of the 472 participants in the study were taking statins, with 34% at a high ASCVD risk, only 3 patients (1.7%) received high-intensity statins [31]. Both Sri Lankan studies emphasize that even though the majority of eligible diabetic patients were prescribed statins as either a primary or secondary preventive strategy, these prescriptions did not align with the individual ASCVD risk level. As a result, more patients with high ASCVD risk remain undertreated, and therefore the expected protection from ASCVD from intensive statin therapy for them cannot be expected. These findings underscore the importance of implementing a mandatory health care policy aimed at preventing ASCVD across the nation.

Subgroup analysis of 683 patients on their mean LDL-C levels and achievements of recommended LDL targets across the three ASCVD risk categories revealed that nearly one-third (236/ 683) of participants have not achieved the recommended LDL-C target. Finding that 50% of the patients with high ASCVD risk fail to meet the LDL target is a greater public health concern. A study with 1196 T2DM patients who have used high-intensity statins has led LDL-C target achievement in only 19.3% of high-risk groups [40]. An Italian study with 4142 T2DM patients revealed that in primary prevention, none of the patients at very high ASCVD risk had LDL-C <  70 mg/dL [41].

The finding of higher mean LDL-C level in patients with high ASCVD risk treated with high intensity statins compared to the group with low ASCVD risk treated with high-intensity statins questions the efficacy of currently practiced high-intensity lipid-lowering therapy in Sri Lankan setting. One possibility of this finding could lie in the prescription of comparatively lower doses of high-intensity statin used for patients in this region. Evidence from clinical trials recommended high-intensity statin dose as atorvastatin 40 mg and rosuvastatin 20 mg. However, most South Asian guidelines recommend half the dose used in the clinical trials with the postulate that South Asian ethnicity, compared to the Western population responds to lower doses of statin. It is possible that the currently used high-intensity dose of statin (20 mg of atorvastatin or 10 mg rosuvastatin) is not effective in reducing the LDL goal in T2DM patients with higher ASCVD risk. A high-intensity statin dose similar to the western region to achieve recommended LDL-C targets in patients with high ASCVD risk is highlighted in this finding. Moreover, a lack of statistically significant difference in mean LDL-C levels across all ASCVD risk categories based on the type of statin prescribed underscores that atorvastatin is comparatively more cost-effective than rosuvastatin, particularly in LMICs such as Sri Lanka.

Concerning the target LDL-C achievement, the significant associations revealed from this study include female gender, poor medication adherence, reduced physical activity, and poor glycemic control. The female gender is well known to be associated with lower use of statins both in patients with and without diabetes. In a matched cross-sectional study of 140,906 patients with T2DM, women without previous CVD showed higher levels of total cholesterol (12.13 mg/dL (0.31 mmol/L); 95% CI =  11.9 − 12.4) and LDL-C (5.50 mg/dL (0.14 mmol/L); 95% CI =  5.3 − 5.7) than men [42]. Analysis of high-risk patients’ target LDL-C achievement revealed that females have a significantly lower probability of reaching LDL-C recommended targets [29]. The other association of non-achievement of recommended LDL-C levels such as poor medication adherence, reduced physical activity, and poor glycemic control possibly reflects the undesirable health habits and suboptimal lifestyle modifications among asymptomatic patients and emphasizes the need for lifestyle modifications for the optimal cardiometabolic risk factor control and cardiovascular health [43–45].

This study highlights several important public health measures useful to reduce CVD burden in a developing Asian country. Although the prescription of statins for primary prevention of ASCVD for patients with T2DM in Sri Lanka is better, the use of high-intensity statins and achievement of recommended LDL-C targets is suboptimal in a majority of patients with moderate-high ASCVD risk. Several barriers may hinder optimal statin prescription in the clinical setting, including the high patient load in busy clinics, a low physician-to-patient ratio, financial constraints, limited physician awareness of updated guidelines, and challenges in patient adherence to prescribed therapies. Education of medical professionals to manage patients with diabetes according to their ASCVD risk is urgently needed. Healthcare practitioners should prescribe statins to diabetic patients based on their assessment of ASCVD risk, and it is recommended that it be made a required healthcare policy. In a busy clinical setting, using these ASCVD risk assessment tools as electronic clinical decision support systems might be a better and more cost-effective way to deal with this problem [46]. Increasing statin use in females without major contraindications and the value of periodic advice and proactive measures to optimize glucose control, increased physical activity and adherence to prescribed medications are the other measures worth implementing in cost-effectively reducing the ASCVD burden.

### Strengths and Limitations

Multi-center study design, enrollment of a sufficient number of patients from tertiary care centers from the most populated five provinces in the country representing all ethnicities, and use of validated tools to assess medication adherence and physical activity patterns are the main strengths of this study. However, there are several noteworthy limitations to our study. Recruitment method (systematic random sampling with a fixed skip interval) of patients may introduce a potential selection bias for the study and it was identified as a limitation. Recruiting patients solely from tertiary care facilities where specialist physicians are present and most resources, including medications, are offered to the public at no cost may not accurately reflect the true burden of the disease nationwide, particularly in the primary care setting, thereby influencing the generalizability of the findings. Although the research identified several factors associated with ASCVD risk levels, statin use, and achieving LDL-C targets in diabetes patients, its dependence on a cross-sectional study limits the capacity to draw causal inferences. However, all the enrolled patients regularly attended clinics for follow-ups, and their medical records comprehensively documented all pertinent information. Therefore, even though the study collected some data retrospectively, we maintain that this did not undermine the validity of the findings. Therefore, the absence of a longitudinal study in this research does not pose a significant methodological concern. Other medications given to diabetes patients with multimorbidity, like hydrochlorothiazide, may have limited effects on LDL-C. Non-collection of such information is considered as a limitation of this study. The Morisky medication adherence scale was employed to assess medication compliance, a tool that is validated for the Sinhala-speaking population; nonetheless, the limitations of the study must also consider the complete reliance on patients’ perspectives concerning their adherence to medication.

## Conclusion

This multicenter study revealed that despite over 80% of patients with diabetes being prescribed statins for primary prevention of ASCVD in the tertiary care setting, only about one-third with moderate-high ASCVD risk received high-intensity statins. Even with high-intensity statin therapy, nearly half of the statin-treated patients failed to meet LDL-C targets. The results highlight the need for a mandatory health policy that directs the prescribers to prescribe statins based on individual ASCVD risk in diabetes patients, in order to effectively address the growing cardiovascular burden in this population.
